# EMMPRIN/CD147 is a novel coreceptor of VEGFR-2 mediating its activation by VEGF

**DOI:** 10.18632/oncotarget.2870

**Published:** 2015-03-23

**Authors:** Farah Khayati, Laura Pérez-Cano, Kamel Maouche, Aurélie Sadoux, Zineb Boutalbi, Marie-Pierre Podgorniak, Uwe Maskos, Niclas Setterblad, Anne Janin, Fabien Calvo, Céleste Lebbé, Suzanne Menashi, Juan Fernandez-Recio, Samia Mourah

**Affiliations:** ^1^ INSERM UMR-S 976, Hôpital Saint-Louis, Paris, France; ^2^ Université Paris-Diderot, Sorbonne Paris Cité, Paris, France; ^3^ Assistance Publique-Hôpitaux de Paris, Laboratoire de Pharmacologie-Génétique, Hôpital Saint-Louis, Paris, France; ^4^ Joint BSC-IRB Research Program in Computational Biology, Life Sciences Department, Barcelona Supercomputing Center, Barcelona, Spain; ^5^ Département de Neurosciences, Institut Pasteur, Unité de Neurobiologie Intégrative des Systèmes Cholinergiques, Paris, France; ^6^ Plateforme d'Imagerie, IUH, Hôpital Saint-Louis, Paris, France; ^7^ INSERM U728, Laboratoire de Pathologie, Hôpital Saint-Louis, AP-HP, Paris, France; ^8^ Département de Dermatologie Hôpital Saint Louis, Paris, France; ^9^ CNRS-UMR 7149, Laboratoire CRRET, Créteil, France; ^10^ Université Paris 12, Créteil, France

**Keywords:** VEGFR-2, EMMPRIN/CD147, interaction/activation, coreceptor

## Abstract

EMMPRIN/CD147 is mainly known for its protease inducing function but a role in promoting tumor angiogenesis has also been demonstrated. This study provides evidence that EMMPRIN is a new coreceptor for the VEGFR-2 tyrosine kinase receptor in both endothelial and tumor cells, as it directly interacts with it and regulates its activation by its VEGF ligand, signalling and functional consequences both *in vitro* and *in vivo*. Computational docking analyses and mutagenesis studies identified a molecular binding site in the extracellular domain of EMMPRIN located close to the cell membrane and containing the amino acids 195/199. EMMPRIN is overexpressed in cancer and hence is able to further potentiate VEGFR-2 activation, suggesting that a combinatory therapy of an antiangiogenic drug together with an inhibitor of EMMPRIN/VEGFR-2 interaction may have a greater impact on inhibiting angiogenesis and malignancy.

## INTRODUCTION

Angiogenesis is a key component of the tumor microenvironment, essential for tumor growth and invasion. Among the angiogenic regulators, vascular endothelial growth factor (VEGF) is known to be the major actor not only in endothelial cells but also in tumor cells, promoting survival, proliferation, apoptosis and migration [1].

VEGF exerts its angiogenic effects by binding to its main receptor (VEGFR-2) or KDR [1, 2]. Binding initiates receptor dimerization which subsequently activates the intracellular tyrosine kinase domains [2]. Active VEGFR-2 then initiates several downstream cell signalling pathways, including stress-activated protein kinase 2/p38 MAP kinase, phosphatidylinositol-f3 kinase, Focal Adhesion Kinase (FAK) and AKT, which culminate in endothelial cell migration, proliferation and vessel formation. The extracellular domain of VEGFR-2 consists of 7 Ig-homology domains. The first 3 domains were shown to mediate ligand binding whereas the membrane proximal domains are involved in ligand-induced receptor dimerization [3–5].

EMMPRIN/CD147, a membrane spanning glycoprotein particularly known as a regulator of matrix degrading proteinases such as MMPs and uPA, has been more recently shown by us and by others [6–8] to be implicated in angiogenesis via the regulation of VEGF expression. Our more recent reports described the concomitant regulation by EMMPRIN of VEGF receptor VEGFR-2 in both endothelial cells and tumor cells, in a mechanism mediated by HIF-2 alpha [9] thus increasing respectively angiogenesis and malignancy. It was also shown to have several other malignancy promoting functions including tumor cell invasion, survival and anchorage-independent growth [10]. Indeed, EMMPRIN has been greatly implicated in malignancy as it is highly expressed in most cancer tissues and its expression often correlates with tumor progression [11–14].

EMMPRIN belongs to the immunoglobulin (Ig) superfamily and is composed of two C2-like immunoglobulin extracellular domains, a transmembrane domain and a short cytoplasmic domain [15]. The extracellular region, which contains three conserved N-glycosylation sites that are variably glycosylated, has been implicated in EMMPRIN self association [16], while the first Ig domain within this region is required for counter-receptor activity involved in MMP induction [17]. The highly conserved transmembrane domain and the short cytoplasmic domain are thought to be implicated in interactions between EMMPRIN and other molecular partners within the membrane. In particular, EMMPRIN was shown to interact with integrins α3β1 and α6β1, enhancing the adhesion and spreading of the cell to the ECM [18] and to caveolin-1 in lipid rafts leading to a decrease in EMMPRIN cell surface self association [19]. The ability of EMMPRIN to associate with different proteins was suggested to determine the different cellular functions attributed to it, although the nature of such interactions and their involvement in signal transduction has not yet been determined.

Our present study uncovered, in both endothelial cells and tumor cells, a unique mechanism of action showing that a direct interaction of EMMPRIN with VEGFR-2 on the plasma membrane is required for VEGF-induced VEGFR-2 activation and downstream signaling. These findings have to be taken in account in tumor angiogenesis combinatorial therapy development.

## RESULTS

### EMMPRIN/CD147 interacts with VEGFR-2 in its non-phosphorylated and phosphorylated forms in endothelial and tumor cells *in vitro* and *in vivo*

**Figure 1:**
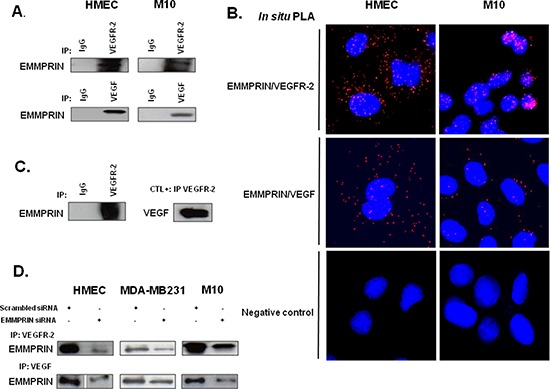
EMMPRIN/CD147 interacts with VEGFR-2 and VEGF in endothelial and tumor cells **(A)** VEGFR-2 and VEGF from HMEC and M10 cell lysates were immunoprecipitated (IP) with anti-VEGFR-2 and anti-VEGF antibody respectively; western blotting was performed using anti-EMMPRIN antibody. Non immune IgG was used as controls. Representative blots of three independent experiments are shown. **(B)** *In situ* Proximity ligation assay (PLA) detection of EMMPRIN-VEGFR-2 and EMMPRIN-VEGF heterodimers (red dots). Negative controls without primary antibody are also shown. Nuclei were stained with DAPI (blue), magnification x 63. Representative images of three independent experiments are shown. **(C)** Direct interaction between the recombinant EMMPRIN and the recombinant VEGFR-2 *in vitro*. VEGFR-2 was first incubated with protein G beads prior to the addition of the recombinant EMMPRIN. Bound proteins were subsequently analyzed by Western blotting. Non-immune IgG served as a negative control and interaction between VEGF and VEGFR-2 served as a positive control. **(D)** Cells (HMEC, MDA-MB-231 and M10) were transfected for 24 hours with EMMPRIN siRNA or scrambled control siRNA at 33nmol/L concentration, and then subjected to IP assays using antibodies against VEGFR-2 and VEGF. Western blotting was performed using anti-EMMPRIN antibody. Representative blots of three independent experiments are shown.

The potential interaction between EMMPRIN and VEGFR-2 was investigated by immunoprecipitation (IP) assays in endothelial cells HMEC and melanoma cells M10. Complex formation was identified by the immunoprecipitation of either VEGFR-2 or VEGF followed by EMMPRIN immunoblotting (Figure 1A). IgG was used as a negative control. The fluorescent red spots observed using *in situ* proximity ligation assay (PLA) (Figure 1B) and confocal microscopy, a method which highlights protein/protein closely colocalized in cells, confirmed the proximity between EMMPRIN and VEGFR-2, and to a lesser extent between EMMPRIN and VEGF, at the cell surface.

To further investigate whether EMMPRIN interacts directly with VEGFR-2 in a cell-free system, we performed pull-down assays using recombinant EMMPRIN and recombinant VEGFR-2. Our results show that VEGFR-2 bound specifically to EMMPRIN and to the same extend as to VEGF, used as a positive control (Figure 1C).

**Figure 2:**
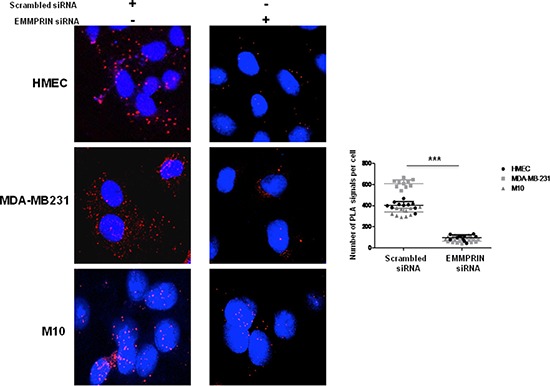
EMMPRIN silencing decreases EMMPRIN/VEGFR-2 interaction in endothelial and tumor cells Cells were transfected with EMMPRIN siRNA or scrambled siRNA prior to *in situ* PLA for EMMPRIN-VEGFR-2 interaction. Cell nuclei were stained with DAPI (blue), magnification x 63. The detected dimers (EMMPRIN/VEGFR-2) are represented as red dots. Representative images of three independent experiments are shown. Quantification of PLA signals was performed on ~150 transfected cells per condition in three independent experiments; mean PLA signal/cell ± SD are plotted. ***P ≤ 0.0001.

The specificity of EMMPRIN/VEGFR-2 interaction was demonstrated by the decrease in the immunoprecipitated (IP) complex when EMMPRIN expression was silenced using siRNA strategy (Figure 1D). This was confirmed by PLA assay showing a large decrease in the number of red dots of cells transfected with EMMPRIN siRNA in both endothelial and tumor cells compared with its corresponding scrambled siRNA (Figure 2). Similar results were obtained with BLM melanoma cells (not shown).

**Figure 3:**
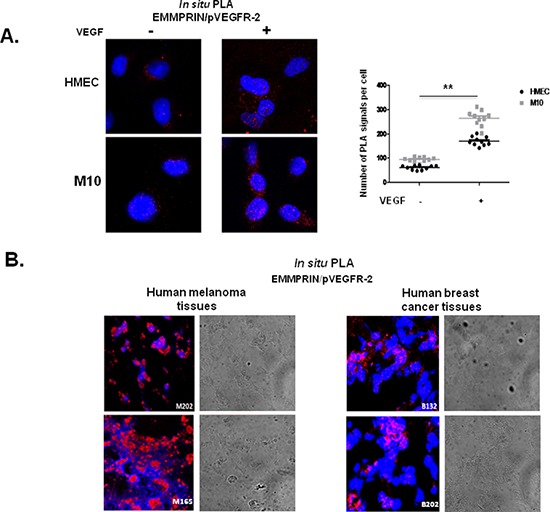
EMMPRIN interacts with pVEGFR-2 *in vitro* and *in vivo* **(A)** EMMPRIN interacts with pVEGFR-2 in HMEC endothelial cells and M10 tumor cells. *In situ* PLA for EMMPRIN/pVEGFR-2 was performed after VEGF stimulation (5 minutes, 50 ng/ml); red dots represent EMMPRIN-pVEGFR-2 interaction; nuclei are stained with DAPI (blue). Representative images of three independent experiments are shown. Quantification of PLA signals was performed on ~150 transfected cells per condition in three independent experiments; mean PLA signal/cell ± SD are plotted. **P ≤ 0.001. **(B)** EMMPRIN interacts with pVEGFR-2 in human cancer tissues. *In situ* PLA detection of EMMPRIN and pVEGFR-2 interaction in human melanoma tissues (M202 and M165) and in human breast cancer tissues (B132 and B18) using antibodies against EMMPRIN and pVEGFR-2. Nuclei were stained with DAPI (blue); phase contrast indicates cell contour (grey); the panels show high magnification (x 40) to clearly visualize the PLA spots representing heterodimers. Representative photos of three independent experiments are shown.

We have next shown that EMMPRIN also interacted with the active form of VEGFR-2 and this interaction was enhanced after VEGF treatment of endothelial as well as melanoma cells. EMMPRIN/pVEGFR-2 heterodimers are visualized by PLA red dots in Figure 3A. Importantly, intense clustering pattern of these EMMPRIN/pVEGFR-2 heterocomplexes were also observed in human breast cancer (*n* = 11) and melanoma (*n* = 15) tissues (Figure 3B) demonstrating the implication of EMMPRIN/pVEGFR-2 interactions *in vivo*.

**Figure 4:**
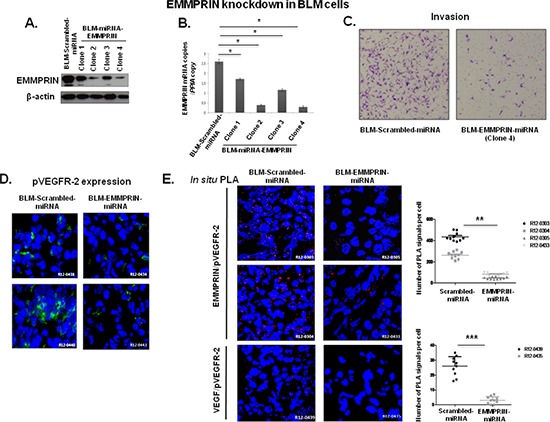
EMMPRIN knockdown decreases EMMPRIN/pVEGFR-2 and VEGF/pVEGFR-2 interactions *in vivo* Melanoma cell line BLM was transfected with EMMPRIN-miRNA (BLM-EMMPRIN-miRNA) or scrambled-miRNA (BLM-Scrambled-miRNA). EMMPRIN expression in 4 different clones was analyzed by: **(A)** western blot (Western Blot was performed usinganti-EMMPRIN antibody normalized to actin; representative blots of three independent experiments); **(B)** by qRT-PCR (means of relative expression to the reference gene PPIA of at least 3 independent experiments, error bars refer to 95% confidence intervals; *P < (0.05)) and **(C)** Invasion assay using a modified Boyden chamber was performed with clone 4 BLM-EMMPRIN-miRNA. Representative images of three independent experiments are shown. EMMPRIN/pVEGFR-2 interaction in experimental mouse model using EMMPRIN deficient tumor cells. Melanoma cell line BLM was transfected with EMMPRIN-miRNA (BLM-EMMPRIN-miRNA) or scrambled-miRNA (BLM-Scrambled-miRNA). **(D)** Immunofluorescence analysis of pVEGFR-2 in xenograft tumors from Scrambled-miRNA or EMMPRIN-miRNA BLM cells 5 weeks after injection. Representative images of 10 primary tumors analysed are shown. **(E)** EMMPRIN/pVEGFR-2 and VEGF/pVEGFR-2 interactions in EMMPRIN-silenced xenografts by *in situ* PLA. Nuclei were stained with DAPI (blue), magnification x 40. Representative images of 10 primary tumors analysed are shown. Quantification of PLA signals was performed on ~150 transfected cells per condition in three independent experiments; mean PLA signal/cell ± SD are plotted. **P ≤ 0.001;***P ≤ 0.0001.

To investigate the role of EMMPRIN in VEGF/pVEGFR-2 interaction *in vivo*, we generated melanoma BLM cells with stable knockdown of EMMPRIN (EMMPRIN-miRNA) for injection in *nude* mice. The 4 clones of BLM-EMMPRIN-miRNA analyzed showed a decrease in EMMPRIN expression (protein and mRNA) in comparison to BLM-srambled-miRNA. This decrease was greatest in clone 2 and 4 which also correlated with the lowest invasive capacity of these clones; clone 4 was chosen for the *in vivo* studies (Figure 4A, 4B, 4C).

Analysis of tumor xenograft sections showed a decrease in pVEGFR-2 immunostaining and in EMMPRIN/pVEGFR-2 interaction in EMMPRIN knockdown tumors (BLM-EMMPRIN-miRNA), compared to control tumors (BLM-scrambled-miRNA). Importantly, this was associated with a significant decrease in VEGF/pVEGFR-2 interaction (Figure 4D, 4E).

### EMMPRIN is required for VEGF-mediated VEGFR-2 activation and downstream signalling

**Figure 5:**
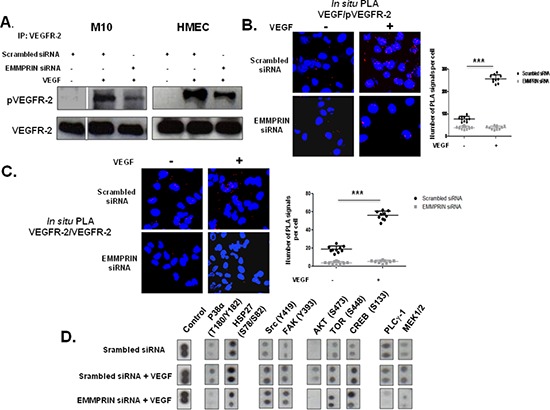
EMMPRIN enhances VEGF-mediated VEGFR-2 activation (phosphorylation and homodimerization) in EMMPRIN silenced HMEC and M10 cells Phosphorylation of VEGFR-2 by VEGF (5 minutes, 50 ng/ml) was assessed by: **(A)** VEGFR-2 IP followed by immunoblotting for pVEGFR-2 and VEGFR-2 used as loading control (representative blots of three independent experiments are shown), and **(B)** *In situ* PLA showing VEGF/pVEGFR-2 interaction (red dots). Quantification of PLA signals was performed on ~150 transfected cells per condition in three independent experiments; mean PLA signal/cell ± SD are plotted. ***P ≤ 0.0001 (magnification x 63). **(C)** *In situ* PLA detection of VEGFR-2 homodimers in HMEC endothelial cells. Nuclei were stained with DAPI (blue), magnification x 63. **(D)** VEGF-induced downstream signalling by Phospho-Proteome profiling of EMMPRIN silenced HMEC cells. Total cell lysates (300μg) were incubated with Human Phospho-Kinase Array membranes (containing 43 different kinases) (R&D systems) and developed by chemiluminescent system. Representative dots of selected kinases are shown.

We next investigated the potential role of EMMPRIN in the activation of VEGFR-2 by its VEGF ligand. Immunoprecipitation of VEGFR-2 followed by immunoblotting with pVEGFR-2 antibody have shown that EMMPRIN knockdown by siRNA decreased VEGFR-2 phosphorylation mediated by VEGF, in both endothelial and tumor cells (Figure 5A). Furthermore, PLA experiments have shown that the reduced activation of VEGFR-2 observed with EMMPRIN inhibition was associated with a decrease in both VEGF/pVEGFR-2 interaction (Figure 5B) and VEGFR-2 homodimerization (Figure 5C) (the decrease was even greater in the presence of VEGF), demonstrating the importance of EMMPRIN in VEGFR-2 phosphorylation mediated by VEGF (Figure5B). Phospho-Proteome Profiler Array analysis showed that certain VEGF-induced downstream signals were inhibited upon EMMPRIN silencing (Figure 5D). Of interest, the activation of p38 and its downstream HSP27 involved in migration were impaired, but not that of the kinases FAK or Src. In addition, the PLCγ-1/MEK1/2 pathway involved in cell proliferation was also affected. These findings suggest a role of EMMPRIN in VEGF signalling.

The functional consequence of this regulation was demonstrated by a decrease in cell migration observed in EMMPRIN siRNA transfected cells that was not restored after VEGF treatment.

**Figure 6:**
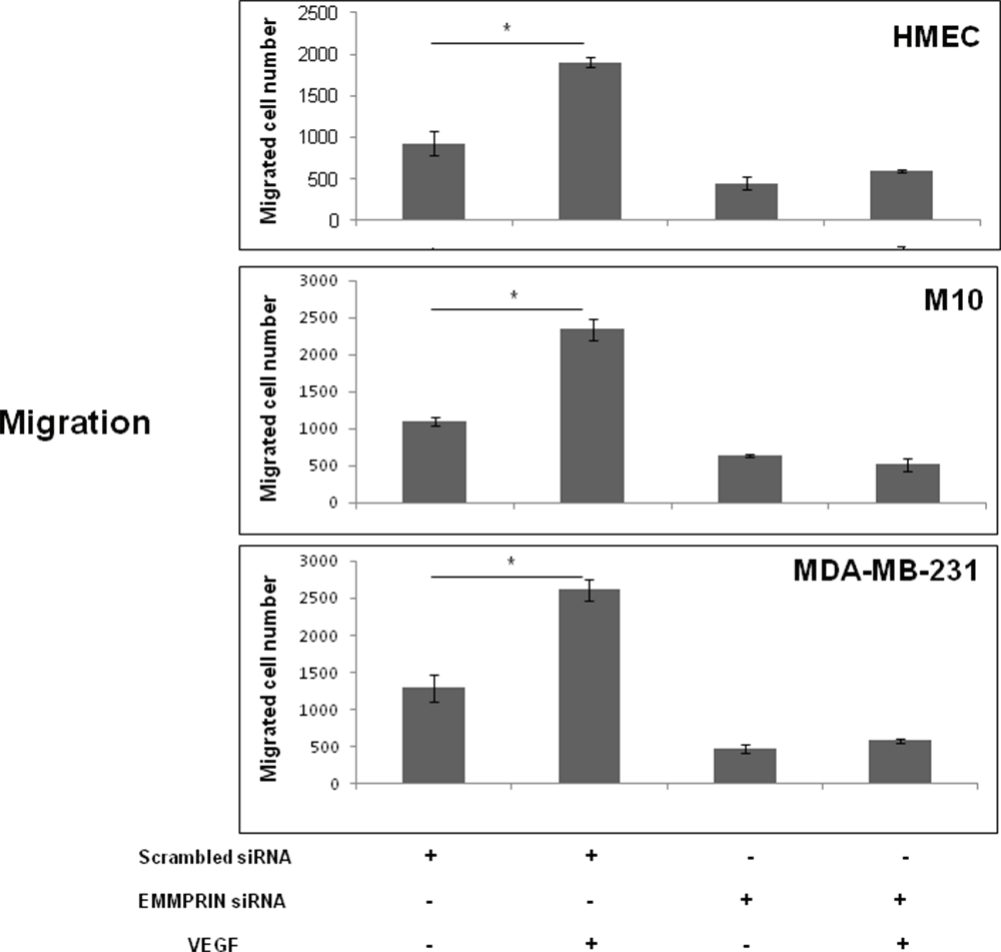
EMMPRIN is required in VEGF-induced VEGFR-2 cell migration Cell migration was determined using a transwell system. EMMPRIN siRNA transfected cells (HMEC, MDA-MB-231 and M10) were seeded in 24-well/insert of Boyden chambers and treated with VEGF (50 ng/ml). After 24 hours of incubation, cells were fixed, stained with Diff-Quick and counted under a microscope. Columns indicate means of 3 independent experiments carried out in triplicate; and bars, SD *P < 0.05.

Cell migration was investigated using modified Boyden chamber assays. Migration of HMEC, M10 and MDA-MB-231 cells in which EMMPRIN was inhibited with siRNA was measured in the presence or not of VEGF. As shown in Figure 6, EMMPRIN silencing greatly inhibited cell migration stimulated by VEGF in the studied models.

### A molecular model for EMMPRIN/VEGFR-2 direct interaction

**Figure 7:**
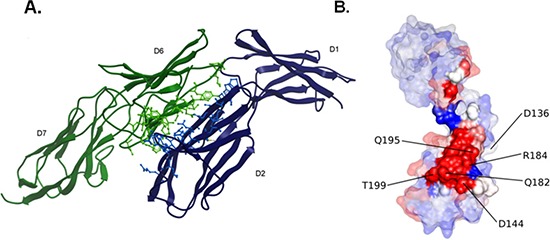
EMMPRIN/VEGFR-2 docking model **(A)** Best-energy docking model for the interaction between EMMPRIN monomer (blue) and VEGFR-2 D6-D7 model (green). Interface residues are shown in ball & stick. **(B)** Surface representation of EMMPRIN monomer residues, colored according to their electrostatic contribution to the VEGFR-2 D6-D7 binding energy (highest contributors in red). Interface residues are highlighted.

The above findings indicate that EMMPRIN interacts with VEGFR-2 to enhance its activation by VEGF, therefore we aimed to build a structural model for EMMPRIN/VEGFR-2 complex that could explain the experimental results. Modeling this system presented several major challenges such as: (i) it was not known whether EMMPRIN interacts with VEGFR-2 as monomer, dimer or oligomer; (ii) the interacting molecules are expected to have significant interdomain flexibility; and (iii) it was not known which VEGFR-2 domain could be involved in the interaction. Therefore we performed a comprehensive modeling strategy using all the available structural information for the possible interacting domains (see Methods). The best docking model was obtained by using one of the EMMPRIN monomers from the x-ray structure and a two-domain construct of VEGFR-2 D6-D7 domains. Interestingly, the lowest-energy binding mode obtained by docking simulations was compatible with membrane binding, and had the majority of interactions between EMMPRIN D2 and VEGFR-2 D6 domains (Figure 7).

### Site-directed mutagenesis confirm EMMPRIN/VEGFR-2 interaction model

The contribution of hot spots residues to EMMPRIN/VEGFR-2 interaction according to the above described model was examined by computational analysis. The EMMPRIN/VEGFR-2 interface according to this model is highly electrostatic (Figure 7B). Based on the model, and considering the residues with highest electrostatic binding energy that were not involved in important intra-domain interactions, the following EMMPRIN mutants were constructed in order to validate the binding interface site: D144A, Q182A, R184A, Q195A, T199A (Figure 7). We also generated Q182A/R184A and Q195A/T199A double mutants (see Methods). D136A was defined as a negative control, since according to the model this residue should not be involved in the interaction.

**Figure 8:**
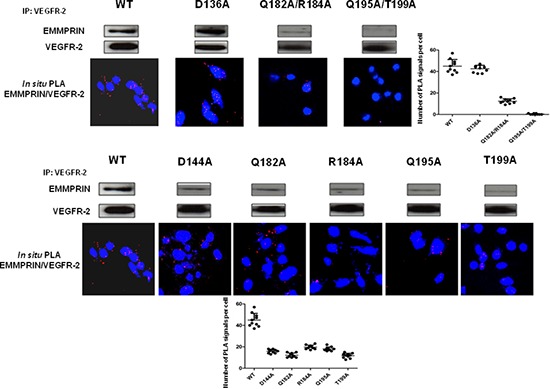
EMMPRIN amino acid residues 195–199 are required for EMMPRIN/pVEGFR-2 interaction EMMPRIN/VEGFR-2 interaction in BLM EMMPRIN-deficient cells transfected with EMMPRIN simple and double mutant constructs, control D136A and WT. After VEGFR-2 pull-downs, interaction with EMMPRIN was analyzed by Western blotting. Representative blots of three independent experiments are shown. *In situ* PLA using confocal microscopy shows red fluorescent spots which denote very close localization between EMMPRIN and pVEGFR-2. Fluorescence was markedly decreased with the double mutant Q195A/T199A. Nuclei were stained with DAPI (blue), magnification x 63. Representative images of three independent experiments are shown. Quantification of PLA signals was performed on ~150 transfected cells per condition in three independent experiments; mean PLA signal/cell ± SD are plotted.

We first transfected BLM-EMMPRIN-miRNA cells with EMMPRIN full length cDNA (wide type: WT) or mutated on the following residues: D136A, D144A, Q182A, R184A, Q195A, T199A, Q182A/R184A and Q195A/T199A. EMMPRIN/VEGFR-2 binding was evaluated by immunoprecipitation using an antibody directed against VEGFR-2 (Figure 8). Results show that both the single and the double mutants reduced EMMPRIN/VEGFR-2 binding to a varying degrees but the greatest reduction was observed with the double mutant Q195A/T199A pointing to the importance of this site in the interaction. By contrast, the negative control D136A mutant had no detectable effect on EMMPRIN/VEGFR-2 binding.

**Figure 9:**
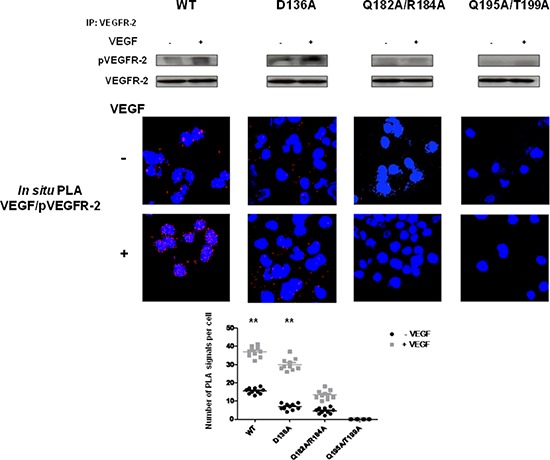
EMMPRIN amino acid residues 195–199 are required for VEGF-mediated VEGFR-2 activation VEGF-mediated VEGFR-2 phosphorylation in BLM EMMPRIN-deficient cells transfected with EMMPRIN double mutant constructs, control D136A and WT. VEGFR-2 phosphorylation by VEGF (5 min) was analyzed by VEGFR-2 IP followed by immunoblotting for pVEGFR-2 and VEGFR-2. Representative blots of three independent experiments are shown. *In situ* PLA was performed to identify VEGF/pVEGFR-2 interaction (red dots) with and without VEGF treatment. Nuclei are stained with DAPI (blue), magnification x 63. Representative images of three independent experiments are shown. Quantification of PLA signals was performed on ~150 transfected cells per condition in three independent experiments; mean PLA signal/cell ± SD are plotted. Comparing PLA signals between VEGF treated and non-treated showed significant difference for WT and control conditions; **P ≤ 0.001.

The role of these EMMPRIN residues on the activation of VEGFR-2 by its ligand VEGF was investigated by studying the binding behaviour of the EMMPRIN mutants towards VEGF-induced VEGFR-2 activation. Figure 9 shows a total inhibition of pVEGFR-2 activation by VEGF with the double mutant Q195A/T199A while the other single mutants had lower effects (Figure S1). This is consistent with the VEGF/pVEGFR-2 interaction results obtained by *in situ* PLA.

Altogether, our results uncovered a novel mechanism by which EMMPRIN regulates VEGFR-2 activation by direct binding, modulating its downstream signalling and functional consequences.

## DISCUSSION

EMMPRIN/CD147 has been reported to play crucial roles not only in matrix proteolysis and tumor invasion but also in angiogenesis [8]. We hypothesized that a possible link between EMMPRIN and VEGFR-2 may exist since both these membrane receptors localized on endothelial and tumor cell surface are involved in common functional properties, notably angiogenesis. In this study, we uncovered a novel function of EMMPRIN as a coreceptor of VEGFR-2, as it directly interacts with it and regulates its activation, signalling and functional consequences. Furthermore, in both endothelial and tumor cells, EMMPRIN enhanced VEGF-induced VEGFR-2 phosphorylation, downstream signalling of the VEGF-induced pathway, and consequently cell migration. Our results show that EMMPRIN/VEGFR-2 interaction involves a binding site located in the extracellular domain of EMMPRIN which contains the amino acids 195/199 located very close to the cell membrane, since mutating this site blocked the interaction. In addition, our *in vivo* studies showed that VEGF/pVEGFR-2 interaction is significantly impaired in mice injected with EMMPRIN-miRNA transfected BLM.

It is interesting to note that high expression of EMMPRIN in human renal cancer was reported to be involved in sunitinib (VEGFR inhibitor) resistance [20]. As EMMPRIN is highly expressed in cancer its interaction with VEGFR-2 may represent one underlying mechanism of this resistance.

**Figure 10:**
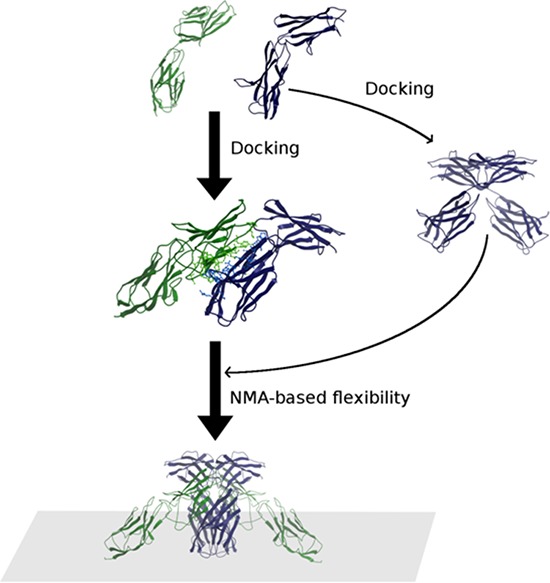
Scheme of the modeling procedure followed in this work The final models were obtained by a combination of EMMPRIN (blue)/VEGFR-2 D6-D7 (green) docking, EMMPRIN/EMMPRIN docking and NMA-based conformational search. Those models compatible with the membrane attachement were selected.

In order to determine whether EMMPRIN/VEGFR-2 binding could explain the enhancement in VEGF-mediated VEGFR-2 dimer formation and VEGFR-2 activation by EMMPRIN, we explored the possible oligomerization state of EMMPRIN when interacting with VEGFR-2 in our model. It has been reported that EMMPRIN can dimerize in cis (both monomers in the membrane of the same cell), through the domain D1, but the structure of the dimer is not known. Therefore, we modeled the dimer of EMMPRIN extracellular domains by docking two monomers from the x-ray structure (see Methods). Interestingly, the lowest-energy docking solution is symmetric and would be compatible with membrane attachment (Figure 10). It should be noted that it was impossible to find a dimer conformation that fully satisfied the recently reported mutational data on EMMPRIN dimerization in solution, which suggests that membrane attachment imposes additional structural restraints to EMMPRIN dimerization [21].

**Figure 11:**
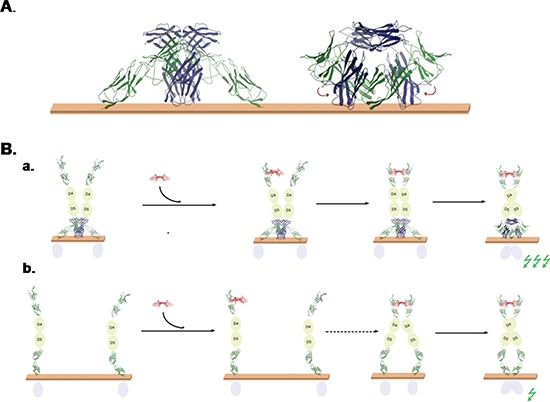
Models of EMMPRIN/VEGFR-2 interaction **(A)** Model of the interaction of EMMPRIN (blue) and VEGFR-2 (green) on the membrane, based on our EMMPRIN/VEGFR-2 D6-D7 docking models, EMMPRIN dimer docking model, and inter-domain NMA-based conformational search. With a small rearrangement of VEGFR-2 D7 domains, this model is compatible with D7/D7 dimer x-ray structure. **(B)** Proposed models for the role of EMMPRIN in VEGF-mediated VEGFR-2 activation. (a) According to EMMPRIN/VEGFR-2 model, EMMPRIN could recruit VEGFR-2 dimers on the membrane surface, which can facilitate binding of VEGF (red) to two VEGFR-2 monomers and hence favour D7/D7 orientation suitable for activation of VEGFR-2 intracellular domains (activation of intracellular signal is represented by a green flash). (b) In the absence of EMMPRIN, VEGFR-2 monomers would be more spread on the membrane surface, so VEGF binding to two VEGFR-2 monomers (second step, marked by a dashed arrow) is less likely and therefore activation of intracellular signal would be smaller.

We combined the above described models obtained for EMMPRIN/VEGFR-2 complex and EMMPRIN dimer, allowing interdomain flexibility with NMA (see Methods), and found many possible rearrangements that are compatible with membrane attachment (see an example in Figure 11A). Interestingly, with a small interdomain rearrangement, the D7 domains could form a dimer as in VEGFR-2 D7 x-ray structure, keeping compatibility with membrane attachment (Figure 11B).

The model shown in Figure 7A for EMMPRIN/VEGFR-2 interaction suggests that EMMPRIN can stabilize a VEGFR-2 dimer in which D7 domains are not in the expected proximity to activate VEGFR-2 intracellular domain. This is compatible with our findings that EMMPRIN can dimerize VEGFR-2 (Figure 5C) but cannot activate it by itself (Figure 5A). However, we also found that EMMPRIN enhances VEGF-mediated VEGFR-2 dimerization and thus activation of intracellular signalling (Figure 5A, 5C). A possible model for this is shown in Figure 11B. When EMMPRIN is present, it can help to recruit VEGFR-2 molecules and form dimers, so when VEGF is added, its probability of binding two VEGFR-2 monomers increases. The binding of VEGF to two VEGFR-2 monomers will facilitate the D7 domains to form a dimer so that the intracellular domains can adopt a suitable orientation that triggers auto-phosphorylation and thus activation of the intracellular signalling (Figure 11Ba). However, in the absence of EMMPRIN, unligated VEGFR-2 molecules are not necessarily located in the proximity of each other and therefore VEGFR-2 dimer formation after VEGF binding, although possible, would be a limiting step that could make the intracellular signalling activation less efficient (Figure 11Bb).

Taken together, these results provide evidence that EMMPRIN is a novel coreceptor of VEGFR-2. EMMPRIN plays a central role in its activation not only in angiogenesis but also in increasing tumor cells malignant properties mediated by VEGFR-2. This should have implications in the design of new strategies to inhibit VEGFR-2 activation. Several innovative antiangiogenic drugs have recently been developed. Doxazosin, an hypertension drug was shown to decrease VEGFR-2/Akt/mTOR signalling and to exert antitumor effects in an animal model [22]. Beside such monotherapy approach, a combinatory strategy using, for example, a dual EGFR inhibition together with anti VEGF treatment have recently shown an improved clinical benefit [23]. In this context, our results propose a novel antiangiogenic approach using an inhibitor of EMMPRIN-VEGFR-2 interaction, which would be expected to be specific for tumor angiogenesis, as EMMPRIN is known to be highly expressed in cancer tissues. Its use in combination with an anti-angiogenic drug may have a greater impact on inhibiting angiogenesis and malignancy.

## MATERIALS AND METHODS

### Cell culture

Human microvascular endothelial (HMEC) cells line derived from dermal microvasculature (T. Lawley, Emory University, Atlanta, GA) were maintained in MCDB-131 medium (Gibco, Invitogen) with 10% fetal bovine serum (FBS) (Invitrogen), 2 ml glutamine (Invitrogen), 10ng/ml endothelial growth factor (Upstate Biotechnology/Millipore), and 1μg/ml hydrocortisone (Sigma-Aldrich). Primary melanoma M10 cells, established from patient primary nodular melanoma were maintained in RPMI medium (Gibco, Invitogen) with 10% FBS, Hepes 1M, pyruvate Nas, and glutamine (Invitrogen). Human breast carcinoma MDA-MB-231 cells were maintained in Dulbecco's modified Eagle's medium (DMEM) (Gibco, Invitogen) with 10% FBS (Invitrogen) and 2ml glutamine (Invitrogen). Melanoma BLM cells (American Type Culture Collection (ATCC Manassas, VA)) were maintained in DMEM containing 4.5 g/l glucose, 10% FBS, 100 U/ml penicillin and 100 mg/ml streptomycin. HEK293T cells (ATCC) were cultured in DMEM medium (Gibco, Invitogen) supplemented with 10% FBS (Invitrogen), 100 U/ml penicillin, 100 mg/ml streptomycin and 2ml glutamine (Invitrogen).

### Immunoprecipitation and western blotting analyses

Cells treated or not with human recombinant VEGF (50 ng/ml; R&D Systems) for 5 minutes at 37°C were harvested, washed with PBS and lysed in extraction buffer (TBS-Nonidet P-40 solution comprising 50mM Tris buffer pH 7.5, 150mM NaCl, 0.5% Nonidet P-40, 5mM NaF and 0.2mM Na3VO4 in the presence of Complete Protease Inhibitor Cocktail (Roche)). For immunoprecipitation, cell lysates were incubated with antibodies against targeted proteins and Protein G-sepharose beads (Sigma). Immunoprecipitated proteins were subjected to sodium dodecyl sulphate-polyacrylamide gel electrophoresis then transferred to Nitrocellulose membranes and probed with anti-EMMPRIN mAb (555961, BD-Pharmingen), anti-VEGF (C-1) mAb (Sc-7269, Santa Cruz), anti-VEGFR-2 rabbit pAb (Sc-504, Santa Cruz-) or anti-pVEGFR-2 (Tyr 1175) rabbit mAb (2478, Cell Signaling). The proteins were visualized with ECL reagent (Pierce), and their expression was normalized relative to total cell lysate protein concentration.

### In situ proximity ligation assay (PLA)

*In situ* PLA was used to assess protein-protein close proximity. Cells grown on 8-well culture slides (Lab-tek chamber slides (Nunc, #154534)), were immediately fixed and subjected to in situ PLA using the Duolink Detection kit (Olink Bioscience, Sweden) according to the manufacturer's instructions. Briefly, after blocking slides were incubated with mouse anti-EMMPRIN (1:250, 555961, BD, Pharmingen), rabbit anti-VEGFR-2 (1:50; Santa cruz), mouse anti-VEGF (1:200; Santa cruz) or rabbit anti-pVEGFR-2 (Tyr 1175) (1:100; Cell Signalling) primary antibodies. PLA minus and PLA plus probes (containing the secondary antibodies conjugated with oligonucleotides) were added. For VEGFR-2 homodimers detection, primary antibody was prepared using the Probemaker kit (OLINK, Bioscience) according to manufacturer's instructions: 1 mg/ml of monoclonal antibody (affinity purified through a protein G column) was independently conjugated to each of a pair of oligonucleotides to generate plus and minus PLA probes. Thereafter, further oligonucleotides are added, allowed to hybridize to the PLA probes, and ligase joins the two hybridized oligonucleotides to a closed circle. The DNA is then amplified (rolling circle amplification), and detection of the amplicons was carried by a fluorescently labeled probe (Detection Kit 563). Protein complexes were visualized in a laser-scanning confocal microscope (Leica-Lasertechnik) as bright fluorescent signals. For PLA analysis of frozen tumor tissues, cryosections were fixed with 4% Paraformaldehyde for 15 min, and in situ PLA assay was performed as described above for cultured cells. Fluorescent and phase contrast images were taken. Negative controls without primary antibody were performed.

### Small interfering RNA transfection

siRNA for EMMPRIN (IDs: 147251 (5′ GCCAAUGCUGUCUGGUUGCtt 3′) and 215973 (5′ GCUACACAUUGAGAACCUUGtt 3′)) or scrambled siRNA oligos (Ambion/Applied-Biosystems, France) were transfected into cells by using the Lipofectamine-2000 (Invitrogen). Cells were then incubated for 24 h prior to treatment with VEGF and were then analyzed by Co-immunoprecipitation, Western Blotting, in situ PLA, cell migration and phospho-kinase array.

### EMMPRIN stable knockdown

In order to knockdown EMMPRIN expression in BLM cell line, lentivirus-based miRNA was used. MicroRNA sequence EMMPRIN-miRNA (5′-TTCATGAGGGCCTTGTCCTCA-3′) targeting human EMMPRIN was selected with Invitrogen Block-iTRNAi Designer software (http://www.invitrogen.com/rnai), and srambled-miRNA (Invitrogen) was used for the negative control [20]. The U6 promoter-miRNA-Ubiquitin promoter-mCherry cassette was cloned into the BamHI and XhoI sites in the lentiviral vector pTK431 [24]. The vector plasmids (either pTK431-EMMPRIN-miRNA or pTK431-scrambled-miRNA), together with the packaging construct plasmid pDNRF and the envelope plasmid pMDG-VSVG, were cotransfected into HEK293T cells to produce the viral particles [24, 25]. The viral titres were determined by p24 antigen measurements (KPL, Lausanne, Switzerland). BLM cells were plated in a 24-well plate at a density of 10.000 cells/well in culture medium. At 60% of confluent, LV-EMMPRIN-miRNA (121 ng/μL of P24) or LV-scrambled-miRNA (97 ng/μL of P24) was added in 100 μl of complete culture medium without FBS. After overnight incubation with the vectors, medium was refreshed and cells were allowed to growth. For determination of transduction efficiencies, transduced cultures were analyzed by cell sorting with a FACS ARIAIII (Becton-Dickinson, San Jose, CA, USA), real-time PCR, Western blotting and invasion assay.

### Real-time quantitative PCR (qRT-PCR)

Total RNA was extracted from BLM-scrambled-miRNA or BLM-EMMPRIN-miRNAcells using Trizol reagent (Invitrogen). RNA quantity and quality were assessed using the Nanodrop-ND-1000 (Nanodrop Technologies, Wilmington). First-strand cDNA was synthesized using a High-Capacity cDNA Archive Kit (Applied-Biosystems) according to the manufacturer's protocol. EMMPRIN primers were specifically designed (Eurogentec, Belgium). Transcript levels were measured by qRT-PCR using Perfect Master Mix-Probe (AnyGenes, France) on LightCycler-480 (Roche) according to the manufacturer's protocol. The transcript levels were normalized to the housekeeping PPIA (peptidylprolylisomerase A) transcripts.

### Immunofluorescence, confocal microscopy

Sections of BLM-Scrambled-miRNA and BLM-EMMPRIN-miRNA derived tumor tissues were fixed and incubated with primary anti-pVEGFR-2 antibody (Cell signaling) followed by Alexa Fluor 488 fluorescently conjugated secondary antibody (Molecular Probes). DAPI was used for nuclear counterstaining. Confocal images were taken with a Leica inverted confocal microscope (Leica Lasertechnik, Heidelberg).

### Animal experiment

Mouse experiments were conducted according to French veterinary guidelines and those formulated by the council of Europe for experimental animal use (L358–86/609EEC). Female 5-week-old nude/c mice (Janvier) were injected subcutaneously with 5 × 10^6^ stably transfected BLM-EMMPRIN-miRNA or BLM-scrambled-miRNA cells (*n* = 10 mice for each cell line). Five weeks later, all mice were sacrificed by cervical dislocation and tumors were resected and stored in liquid nitrogen prior to in situ PLA and immunofluorescence assays.

### Migration and invasion assays

The *in vitro* migration (on uncoated filters) and invasion (on coated filters with matrigel, BD Bioscience) were performed using a modified Boyden chamber [26] in 24-well plates and 8-mm pore filter inserts (BD Bioscience). After 24 h of incubation, cells were fixed, stained with crystal violet 0.5% and counted under a light microscope.

### Human phospho-kinase array

The human phospho-Kinase Array Kit (Proteome Profiler Array, ARY003, R&D Systems) was used to detect relative levels of phosphorylation of 46 kinase phosphorylation sites, according to the manufacturer's instructions, using total cell lysates of EMMPRIN or scrambled siRNA transfected HMEC cells treated or not with 50 ng/ml VEGF. Briefly, cell lysates diluted to 300 μg/mL of protein in a detergent- urea and phosphatase inhibitor-containing solubilizing buffer (R&D Systems) were incubated with the arrays overnight at 4°C. After washing unbound material, membranes were incubated with a cocktail of phosphosite–specific, biotinylated antibodies, and phosphorylated kinases were detected with streptavidin-horseradish peroxidase. Signals were revealed with a chemiluminescent substrate kit (ECL Dura Thermo Scientific, 34076). Independent experiments were performed in duplicates.

### Modelling: general consideration

We modelled EMMPRIN/VEGFR-2 association by using homology-based modeling, computational docking, and conformational sampling by normal mode analysis (NMA) (Figure 10). On the one side, we built a model for VEGFR-2 D6-D7, and we docked it to EMMPRIN x-ray structure. The EMMPRIN/VEGFR-2 docking model was later confirmed by site-directed mutagenesis. We also used docking to build models for EMMPRIN dimerization. The docking models obtained for EMMPRIN/VEGFR-2 and EMMPRIN dimer, in combination with NMA-based sampling, were compatible with membrane attachment, and with D7 x-ray dimers. We tried other docking combinations but could not find any better model.

### Homology-based modeling of VEGFR-2 D6-D7

We used Modeller 8v1 [27] to model the structure of VEGFR-2 D6-D7 domains from 1F97 PDB template structure, with 24% of sequence identity and selected by FUGUE server (http://tardis.nibio.go.jp/fugue/prfsearch.html) [28] as the best homologous topology. The D7 coordinates in the model were replaced by the known x-ray structure (3KVQ PDB). The resulting D6-D7 construct (in particular the linker between D6 and D7 domains) was finally refined by Modeller 8v1.

### Computational docking

Computational docking was performed by combining the 10,000 output solutions from FTDock 2.0 [29] and the 2,000 ones from ZDock 2.1 [30]. The resulting 12,000 solutions were then scored by pyDock [31].

### Energetic analysis of interaction model

The best docking model for EMMPRIN D1-D2/VEGFR-2 D6-D7 was energetically minimized using Tinker (http://dasher.wustl.edu/tinker/). The global binding energy had a clear electrostatic contribution. The binding energy per residue was calculated for this minimized structure using pyDock [31]. Residues with the highest electrostatic contribution were further considered for selecting mutant candidates (Figure 7B).

### NMA-based conformational sampling

We used iMC module from iMOD [32] (with a maximum amplitude of 6Å) for conformational sampling of extracellular EMMPRIN and VEGFR-2 D6-D7 model. With this method, we generated 100 conformations independently for EMMPRIN and VEGFR-2 D6-D7 monomers. These NMA-based EMMPRIN conformations were combined to produced new EMMPRIN dimer models, built based on the main dimer interface described by the EMMPRIN/EMMPRIN docking model. These EMMPRIN dimer models were combined with the NMA-based VEGFR-2 D6-D7 conformations, based on the main interface described by the EMMPRIN/VEGFR-2 D6-D7 docking model. This generated 10,000 (2:2) EMMPRIN/VEGFR-2 D6-D7 models. Finally, when considering cell membrane, we obtained a total of 32 models in which C-term regions from all molecules were located approximately in the same plane (to represent the attachment to the membrane) and with more than 80% of the atoms located in one side of the defined plane (in order to disregard strong steric clashes with the cell membrane).

### Site directed mutagenesis

EMMPRIN residues (Asp144, Gln182, Arg184, Gln195, Asp136 and Thr199) involved in the interaction between EMMPRIN and VEGFR-2 were mutated to Alanine using « Geneart Site-Direct Mutagenesis system » (Lifetechnologies) according to the manufacturer's instructions. The following mutations were made in the PCRII vector containing EMMPRIN full length cDNA (PCRII-EMMPRIN) [10]. Briefly, the mutagenesis reactions were performed using Platinum Taq DNA polymerase (Lifetechnologies), with specifically designed mutagenesis primers (Table 1) and cycling conditions as follows: 37°C for 20 minutes, 94°C for 2 minutes followed by 18 cycles of 94°C for 20 seconds, 57°C for 30 seconds and 68°C for 2.5 minutes; and finally 1 cycle of 68°C for 5 minutes. Each mutagenesis product was transfected into chemically competent DH5α T1R E.coli (Lifetechnologies) and grown at 37°C overnight. Colonies were selected and screened for the correspondant mutation at each site by DNA sequencing. PCRII-EMMPRIN wide type (WT) and mutated were transfected into BLM-EMMPRIN-miRNA cells using the Lipofectamine-2000 (Invitrogen).

**Table 1:** Primer sequences used for mutagenesis

Mutation	Primer Sequence
**D136A**	for: GTGCCACCTGTCACTGCCTGGGCCTGGTACAAG rev: CTTGTACCAGGCCCAGGCAGTGACAGGTGGCAC
**D144A**	for: TGGTACAAGATCACTGCCTCTGAGGACAAGGCC rev: GGCCTTGTCCTCAGAGGCAGTGATCTTGTACCA
**Q182A**	for: ATGGAGGCCGACCCCGGCGCGTACCGGTGCAACGGCAC rev: GTGCCGTTGCACCGGTACGCGCCGGGGTCGGCCTCCAT
**R184A**	for: CGACCCCGGCCAGTACGCGTGCAACGGCACCAG rev: GCTGGTGCCGTTGCACGCGTACTGGCCGGGGTCG
**Q195A**	for: CTCCAAGGGCTCCGACGCGGCCATCATCACGCTC rev: GAGCGTGATGATGGCCGCGTCGGAGCCCTTGGAG
**T199A**	for: CGACCAGGCCATCATCGCGCTCCGCGTGCGCAG rev: CTGCGCACGCGGAGCGCGATGATGGCCTGGTCG
**Q182A/R184A**	for: AGGCCGACCCCGGCGCGTACGCGTGCAACGGCACCA rev: TGGTGCCGTTGCACGCGTACGCGCCGGGGTCGGCCT
**Q195A/T199A**	for: GCTCCGACGCGGCCATCATCGCGCTCCGCGT rev: ACGCGGAGCGCGATGATGGCCGCGTCGGAGC

### Statistical analysis

Data are presented as the mean values ± SD. Mann-Whitney test was used to evaluate differences between groups. Data were considered statistically significantly different for *P* value < 0.05. All statistical tests were two-sided. Analyses were performed using Prism 6 (GraphPad Software Inc, La Jolla, CA).

## SUPPLEMENTARY FIGURE


