# Supplementary material for: EMMPRIN/CD147 is a novel coreceptor of VEGFR-2 mediating its activation by VEGF
Source: Oncotarget. 2015 Mar 23;6(12):9766–80. doi: 10.18632/oncotarget.2870 (PMC4496396; doi:10.18632/oncotarget.2870)
Supplement: Supplementary file 1 [file oncotarget-06-9766-s001.pdf]

SUPPLEMENTARY FIGURE

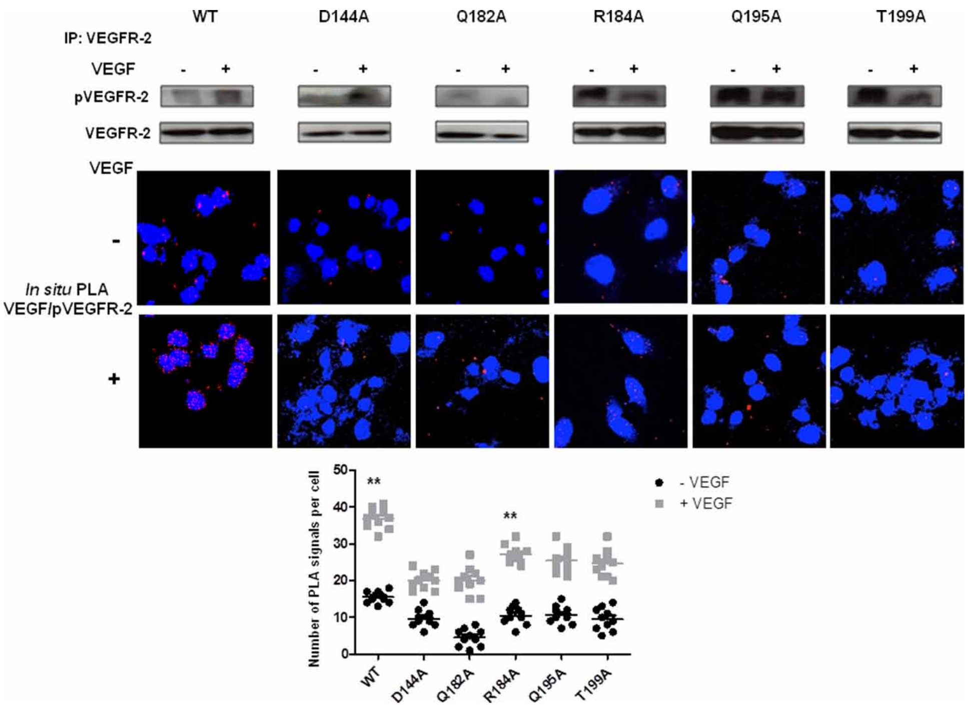

Supplementary Figure S1: VEGF-mediated VEGFR-2 phosphorylation in BLM EMMPRIN-deficient cells transfected with EMMPRIN simple mutant constructs. VEGF-mediated VEGFR-2 phosphorylation in BLM EMMPRIN-deficient cells transfected with EMMPRIN simple mutant constructs and WT. VEGFR-2 phosphorylation by VEGF (5 min) was analyzed by VEGFR-2 IP followed by immunoblotting for pVEGFR-2 and VEGFR-2. Representative blots of three independent experiments are shown. *In situ* PLA was performed to identify VEGF/pVEGFR-2 interaction (red dots) with and without VEGF treatment. Nuclei are stained with DAPI (blue), magnification x 63. Representative images of three independent experiments are shown. Quantification of PLA signals was performed on ~150 transfected cells per condition in three independent experiments; mean PLA signal/cell ± SD are plotted. Comparing PLA signals between VEGF treated and non-treated showed significant difference for WT and control conditions; $**P \leq 0.001$.
